# High prevalence rates for multiple psychiatric conditions among inmates at French Guiana’s correctional facility: diagnostic and demographic factors associated with violent offending and previous incarceration

**DOI:** 10.1186/s12888-018-1742-7

**Published:** 2018-05-29

**Authors:** Mathieu Nacher, Gulen Ayhan, Romain Arnal, Célia Basurko, Florence Huber, Agathe Pastre, Louis Jehel, Bruno Falissard, Vincent About

**Affiliations:** 1 Centre d’Investigation Clinique CIC INSERM 1424, Centre Hospitalier de Cayenne, 97300 Cayenne, French Guiana; 2 Service de Psychiatrie, Centre Hospitalier de Cayenne, 97300 Cayenne, French Guiana; 3 Hôpital de Jour Adultes, Centre Hospitalier de Cayenne, 97300 Cayenne, French Guiana; 4 Unité de Consultations Ambulatoires Carcérales, 97300 Cayenne, French Guiana; 5 CESP- INSERM U1178 Equipe IPSOM, Centre Hospitalier Universitaire de Martinique, BP632, 97261 Cedex fort de France, France; 6 CESP/INSERM U1018 (Centre de Recherche en Epidémiologie et Santé des Populations), 75679 Paris cedex 14, France

## Abstract

**Background:**

French Guiana has the highest incarceration rate among French territories, it is higher than that of Brazil, Colombia or Venezuela. It is well known that mental health problems are over-represented in correctional facilities. Our objectives were to describe the prevalence of various psychiatric conditions and to study factors associated with violence and repeated offenses among arriving detainees at the sole correctional facility of French Guiana.

**Methods:**

The study was cross-sectional. All consenting new adult prisoners incarcerated between 18/09/2013 and 31/12/2014 at the penitentiary centre of French Guiana were included. The Mini International Neuropsychiatric Interview (MINI) was used to screen for psychiatric diagnoses. In addition sociodemographic data was collected.

**Results:**

Overall 647 men and 60 women were included.

The participation rate was 90%.Overall 72% of patients had at least one psychiatric diagnosis (Fig. 2). Twenty percent had three or more diagnoses. Violent index offences were not more frequent among those with a psychiatric diagnosis (crude odds ratio 1.3 (95%CI = 0.9–2), *P* = 0.11.

Multivariate analysis showed that after adjusting for sex and age, psychosis, suicidality and post-traumatic stress disorder were independently associated with violent offences. Generalized anxiety disorder was less likely to be associated with incarceration for violent offences.

Having a history of a previous incarceration was significantly associated with a psychiatric condition in general (any diagnosis) OR = 3 (95%CI = 2–4.3), *P* < 0.0001.

Calculations of the population attributable risks showed that in the sample 31.4% of repeat incarcerations were attributable to antisocial personality disorder, 28.3% to substance addiction, 17.3% to alcohol addiction, 8.7% to depression and 7% to psychosis.

**Conclusions:**

The very high prevalence of psychiatric disorders observed in our sample, and the relative lack of psychiatric facilities, suggest that part of the problem of very high incarceration rate may be explained by transinstitutionalization. Improving psychiatric care in prison and coordination with psychiatric care in the community after release is likely to be important.

## Background

French Guiana is a French overseas territory located between Brazil and Suriname, and thus a part of the European Union. It has the highest GDP per capita on the Latin American continent and therefore attracts numerous immigrants in search of a better life (https://www.insee.fr/fr/statistiques/2011101?geo=DEP-973). However, unemployment is high and much of the population lives in precarious social conditions. The soil is rich in gold and attracts large numbers of illegal gold miners [1]. French Guiana is also a hub for cocaine trafficking towards France and Europe. Drugs are relatively cheap and there are high rates of substance use, impacting on HIV rates [2]. A quarter of families are single parent families, and one in five families includes four children or more, demographic conditions that further increase social vulnerability (https://www.insee.fr/fr/statistiques/2011101?geo=DEP-973).

The natural population growth in French Guiana is 2.45% per year, the second highest in Latin America on a par with Guatemala [3]. This very rapid growth compounds social problems and challenges the French social system, which struggles to keep up in terms of education, health infrastructure, and appropriate housing. These difficult social conditions and the illegal “opportunities to make money” may fuel the high crime rate and lead French Guiana to have the highest incarceration rate (328 per 100,000) among French territories, a rate that is higher than that of Brazil (319 per 100,000), Colombia (231 per 100,000) or Venezuela (173 per 100,000) [4].

As for other government services, the judiciary and penitentiary systems struggle to keep up. The only correctional facility in French Guiana is thus saturated and its overpopulation and poor living conditions have been repeatedly described [5, 6].

It is well known that mental health problems are over-represented in correctional facilities [7, 8]. Psychiatric illnesses often increase the risk of suicide, the risk of death after release from incarceration, and the risk of new offences and reincarceration. Until recently, there had never been any study of the mental health issues of detainees in this particular territory at the crossroad between France and Latin America. A first study showed focused on the risk of suicide and its predictors among arriving detainees [9]. The objective of the present study was to describe the relation between various psychiatric conditions and violence or repeated offenses among arriving detainees at the sole correctional facility of French Guiana.

## Methods

The study was cross-sectional. All consenting new adult prisoners incarcerated between 18/09/2013 and 31/12/2014 at the penitentiary centre of French Guiana at Cayenne were included.

This is slightly different from the study on suicide risk factors [9] because the questionnaire and case record form used was modified after a test phase; we only used data collected with the finalized version of the data collection tool.

Inmates with an assigned legal guardian were excluded in order not to compromise the incarceration procedures for our study because the presence of the legal guardian would have been logistically very difficult given the restricted access to the mental health ward [9].

After incarceration, all new arrivals are seen for physical examination by a doctor of the “Unité de consultation et de soins ambulatoires (UCSA)”, the ambulatory care unit of the prison and then by a psychiatrist or psychiatric nurse in the “Unité fonctionnelle de psychiatrie intra-carcérale (UFPI)” (the psychiatric ward). Patients had a 15 day window after arrival in which they could be included.

In addition to this normal procedure upon admission for the purpose of our study we added the Mini International Neuropsychiatric Interview (MINI). The MINI is a short diagnostic structured interview (DSI) developed in France and the United States to explore 17 disorders according to the Diagnostic and Statistical Manual (DSM)-V diagnostic criteria. The validity and reliability of the MINI has been confirmed in several studies [10, 11]. The MINI is structured to allow administration by non-specialized interviewers for the research of current disorders. It is currently one of the most used psychiatric diagnostic tools [12]. For each mental illness, one or two screening questions rule out the diagnosis when answered negatively. The MINI is thus adapted for epidemiological studies requiring a short but robust tool. The estimated time for the interview is 15 min. The MINI has been translated and validated in 46 languages, including the main languages found in French Guiana: French, English, Portuguese, Dutch and Spanish.

All psychiatrists and nurses performing the MINI were trained in order to correctly use the questionnaire. Socio-demographic questions were added (age, birthplace, residence, languages, presence of a translator, family status, children, siblings, position among siblings, professional situation), history of detention (reason for detention, previous imprisonment) and psychiatric history. A training period preceded our study in order to test the feasibility of the MINI and to familiarize staff with the protocol and to verify that the staff was proficient with the tool before starting the study. Since there were 2 phases with some changes in the ancillary questionnaire, we only analyzed the data collected with the final questionnaire in order to obtain a homogenous data set.

### Inclusion criteria

Incarcerated adults accepting to participate were only included.

### Exclusion criteria

Minors, persons with a legal guardian, or persons refusing to participate were not included.

### Statistical analysis

Descriptive analysis of qualitative and quantitative variables was followed by bivariate analysis in order to identify significant variables for incarceration for violent index offences. Variables were included in a multivariate model for logistic regression in order to identify independent diagnoses at increased risk for incarceration for violent index offences. The modelling strategy was purely exploratory and the variables were retained in the model except obsessive compulsive disorder, anorexia and bulimia which were too rare to be included in a multivariate model (respectively, 15, 5 and 3 persons). Collinearity was tested using the Collin package (STATA, College Station, Texas) and verifying that variance inflation factors were < 4. The Hosmer-Lemeshow goodness-of-fit test was used to test the model.

A similar procedure was used for bivariate and multivariate analysis of variables associated with repeated incarcerations.

Principal component analysis was performed and a loading plot of the different diagnoses was performed.

Stata13 (College Station, Texas, USA) was used.

### Ethical and regulatory aspects

The study was approved by the Ethical committee of Bordeaux (Comité de Protection des Personnes, CPP) (reference number DC 2012/115). The study was also approved by the Ethical committee of INSERM CEEI in 2013 (IRB00003888). Inmates gave informed consent (oral and written) to participate in the study.

## Results

### General results

**Fig. 1:**
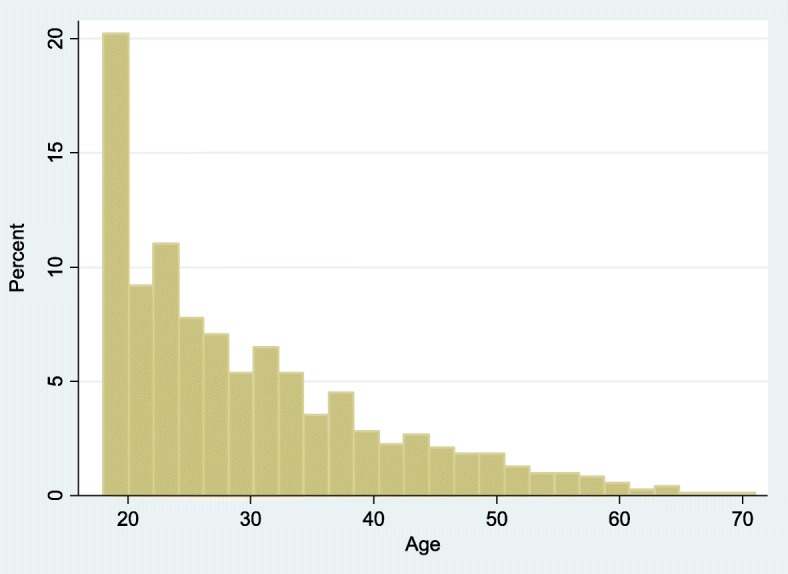
Age of incarcerated persons included in the study at the sole correctional facility of French Guiana

Between September 18^th^2013 and December 31st 2014, 785 new prisoners were registered. The survey participation rate was 90% (707/785) [9]. Overall 647 men and 60 women were included. The mean age was 30 years (SD = 10.7 years) for men and 27.7 years (SD 10 years) for women. Figure 1 shows the over-representation of younger age groups.

#### Birth place and spoken language

Overall 47.8% of persons were born in French Guiana, 3% in other French territories, 15.1% were born in neighboring Suriname, 14.3% were born in Guyana, 10.6% in Brazil, 3.1% in Haiti, and the rest were born elsewhere or missing. 57.3% spoke French, 14% spoke English, 9.1% spoke Portuguese, 7.4% spoke Nengue Tongo, and 7% spoke Haitian Creole.

#### Marital status

Regarding their marital status 48.4% were single, and 41.02% lived in a couple, 6.3% were married and 1% were divorced or widowed. Sixty percent (368/613 respondents) of inmates had parents who were separated (13.3% of non response).

#### Work

Among respondents (92%) to the work status question, 22.4% of men and 48% of women were unemployed; 46% of men and 26% of women declared having odd jobs; and 24.6% of men and 10% of women had a work contract.

#### Trauma

Nearly 2% (11/564 respondents) declared having experienced prior sexual trauma with 18.2% not responding to the question. A third of respondents declared the death of a close family member (233/691 respondents).

#### Incarceration motives

Incarceration resulted from violent offences in 28% of cases (assault 7.9%, homicide without intent 2.2%, intentional homicide 3.5%, rape 4.5%, violence 9.8%) and drugs in 34.4% of cases.

### Psychiatric diagnoses

**Fig. 2:**
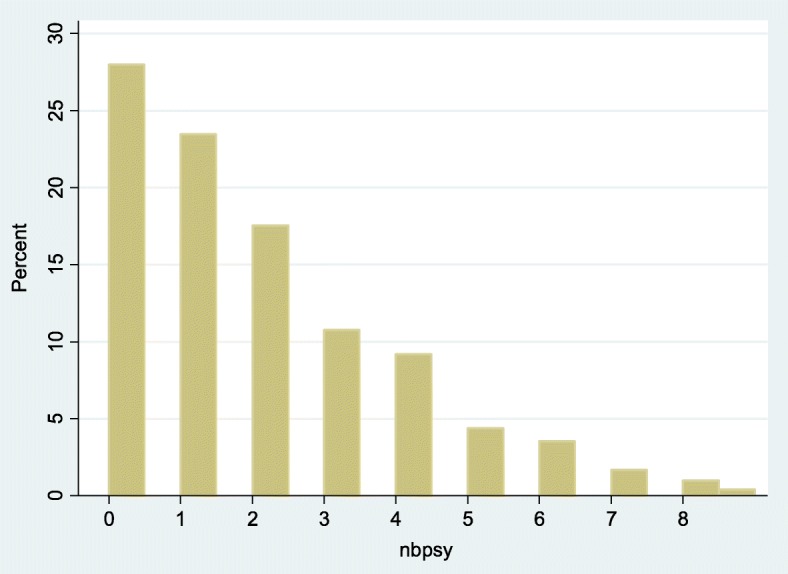
Number of psychiatric diagnoses among inmates incarcerated in the sole correctional facility of French Guiana

Overall 72% of patients had at least one psychiatric diagnosis (Fig. 2). Twenty percent had three or more diagnoses. Violent offences were not more frequent among those with a psychiatric diagnosis (crude odds ratio 1.3 (95%CI = 0.9–2), *P* = 0.11, Median 1 in both groups, *p* = 0.49).

Overall 36 of 51 (70.6%) persons with psychosis also had substance addictions. Conversely, 36 of 235 persons with substance addictions (15.3%) were also psychotic. Among the study population, 307 took cannabis (43%), 58 took crack cocaine (8.2%) and 15 cocaine (2.1%).

**Fig. 3:**
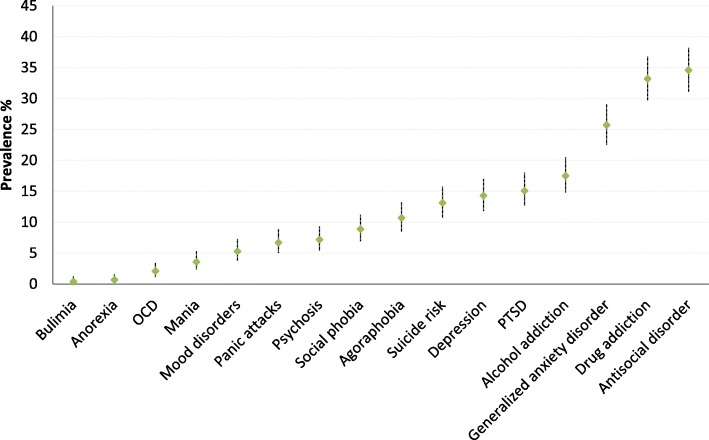
Prevalence and 95% confidence intervals of different psychiatric conditions in Prisoners in French Guiana using the MINI 5.0 screening tool at the time of incarceration

The individual prevalence for individual diagnoses is shown in Fig. 3.

Overall, 2% reported past sexual trauma (95%CI = 1–3.5%) and 5.9% (95%CI = 4.2–8%) reported a family history of psychiatric problems.

#### Principal component analysis

**Fig. 4:**
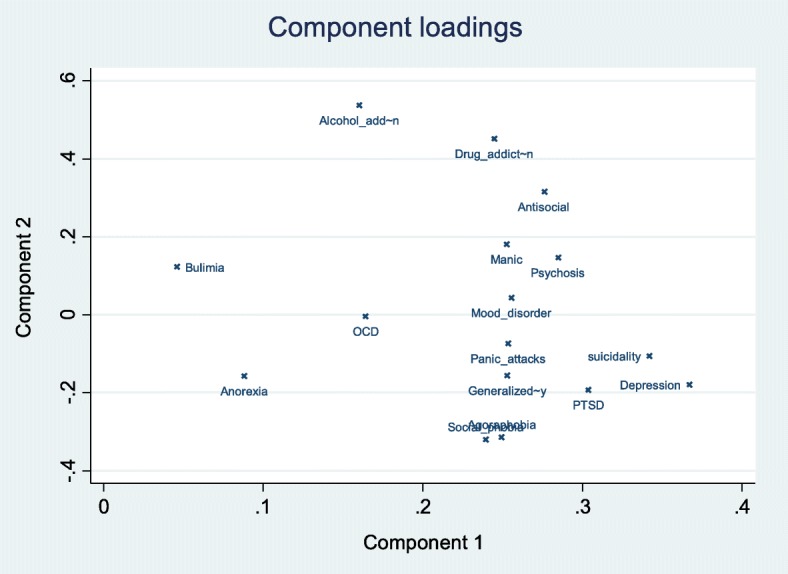
Principal component analysis of the different psychiatric diagnoses among inmates incarcerated in the sole correctional facility of French Guiana

Figure 4 shows a loading plot after the principal component analysis of the different diagnoses obtained by the MINI. A cluster of depressive signs is found on the lower right part, the bottom center is mostly linked to a cluster of neurotic manifestations; the top center cluster pertains to addictions, which lie close to antisocial disorder.

#### Incarceration for violence

Table 1 shows the proportion of patients for listed MINI- diagnoses who had been charged with violent and non-violent crimes. Multivariate analysis showed that after adjusting for sex and age, psychosis, suicidality, and post-traumatic stress disorder were independently associated with violent offences for incarceration. Generalized anxiety disorder was less likely to be associated with incarceration following a violent index offence.

**Table 1:** Variables associated with violent and non violent crimes, bivariate and multivariate analysis

	Total	Incarceration linked to violence N(%)	Incarceration not linked to violence N(%)	Crude odds ratio (95% CI)	Adjusted odds ratio (95% CI), *P*
*Age* in years Mean [SD]	707	29.6 [10.3]	31 [11.2]	1 (0.99–1.02)	1(0.98–1.02), *P* = 0.5
*Sex*					
Male Female	647 60	195 (98.5) 3(1.5)	452(88.8) 57(11.2)	8.2(2.5–26.5)	8 (2.4–26.4), *P* = 0.001
*Antisocial disorder*					
Yes No	245 462	67(33.8) 131 (66.2)	178 (35) 331(65)	0.9 (0.67–1.3)	0.9 (0.6–1.3), *P* = 0.5
*Generalized anxiety disorder*					
Yes No	182 525	39(19.7) 159(80.3)	143(28.1) 366(71.9)	0.6 (0.4–0.9)	0.5 (0.3–0.8), *P* = 0.006
*Psychosis*					
Yes No	51 656	23(11.6) 175(88.4)	28(5.5) 481(94.5)	2.2 (1.2–4)	2.4 (1.2–4.6), *P* = 0.01
*Substance addiction*					
Yes No	235 230	61(30.8) 197 (69.2)	174(34.2) 33(65.8)	0.8(0.6–1.2)	0.7 (0.4–1), *P* = 0.05
*Alcohol addiction*					
Yes No	144 583	39(19.7) 159(80.3)	85(16.7) 424(83.3)	1.2 (0.8–1.8)	1.4 (0.86–2.2), *P* = 0.19
*PTSD*					
Yes No	107 600	40(20.2) 158(79.8)	67(13.1) 442(86.8)	1.7(1.1–2.6)	1.8 (1.08–3), *P* = 0.02
*Social phobia*					
Yes No	63 644	15(7.6) 183(92.4)	48(9.4) 461(90.6)	0.8 (0.4–1.44)	0.7 (0.4–1.5), *P* = 0.4
*Agoraphobia*					
Yes No	76 631	28(14.1) 170(85.9)	48(9.4) 461(90.6)	1.6 (0.96–2.6)	1.6 (0.9–2.9), *P* = 0.1
*Panic attacks*					
Yes No	48 659	15(7.6) 183(92.4)	33(6.5) 476(93.5)	1.2 (0.6–2.2)	1 (0.5–2), *P* = 0.9
*Mania*					
Yes No	26 681	5(2.5) 193(97.5)	21(4.1) 488(95.9)	0.6 (0.2–1.6)	0.4 (0.1–1.2), *P* = 0.1
*Mood disorders*					
Yes No	38 669	11(5.5) 187(94.5)	27(5.3) 482(94.7)	1 (0.5–2.1)	0.9 (0.4–2.2), *P* = 0.9
*Suicidality*					
Yes No	93 614	35(17.7) 163(82.3)	58(11.4) 451(88.6)	1.7 (1.05–2.6)	2.1 (1.2–3.8), *P* = 0.009
*Depression*					
Yes No	101 606	28(14.1) 170(85.9)	73 (14.3) 436(85.7)	1 (0.6–1.6)	0.8 (0.4–1.5), *P* = 0.5

#### Repeat offenders

Overall, 48.6% of inmates had already been previously incarcerated. Having a history of a previous incarceration was significantly associated with a psychiatric condition in general (any diagnosis) OR = 3 (95%CI = 2–4.3), *P* < 0.0001. Table 2 shows the detail by psychiatric diagnosis. Calculations of the population attributable risks showed that in the sample 31.4% of repeat incarcerations were attributable to antisocial personality disorder, 28.3% to substance addiction, 17.3% to alcohol addiction, 8.7% to depression and 7% to psychosis.

**Table 2:** Variables associated with previous incarcerations, bivariate and multivariate analysis

	Total	Previously incarcerated N(%)	First incarceration N(%)	Crude odds ratio (95% CI)	Adjusted odds ratio (95% CI), *P*
*Age* in years Mean [SD]	707	32 [10.5]	28.1 [10.3]	1 (0.99–1.02)	1.04(1.02–1.06), *P* < 0.001
*Sex*					
Male Female	647 60	333(96.8) 11(3.2)	314 86.5) 49(13.5)	4.7(2.4–9.2)	4.3 (2–9.1), *P* < 0.001
*Antisocial disorder*					
Yes No	245 462	162(47.1) 182 (52.8)	83 (22.9) 280(77.1)	3 (2.2–4.1)	2.6 (1.8–3.7), *P* < 0.001
*Generalized anxiety disorder*					
Yes No	182 525	92(26.7) 252(73.3)	90(24.8) 273(75.2)	1.1 (0.8–1.5)	0.8 (0.5–1.3), *P* = 0.4
*Psychosis*					
Yes No	51 656	37(10.7) 307(89.3)	14(3.9) 349(96.1)	3 (1.6–5.7)	1.4 (0.7–2.9), *P* = 0.01
*Substance addiction*					
Yes No	235 472	153(44.5) 191 (55.52)	82(22.6) 281(77.4)	2.7(2–3.8)	1.9 (1.3–2.7), *P* = 0.001
*Alcohol addiction*					
Yes No	124 583	88(25.6) 256(74.4)	36(9.9) 327(90.1)	3.1 (2–4.7)	1.7 (1.1–2.8), *P* = 0.02
*PTSD*					
Yes No	107 600	64(18.6) 280(81.4)	43(11.9) 320(88.1)	1.7(1.1–2.6)	1.3 (0.8–2.2), *P* = 0.3
*Social phobia*					
Yes No	63 644	34(9.9) 310(90.1)	29(8) 334(92)	1.2 (0.74–2.1)	1.2 (0.6–2.3), *P* = 0.6
*Agoraphobia*					
Yes No	76 631	46(13.4) 298(86.4)	30(8.2) 333(91.8)	1.7 (1.05–2.8)	1.1 (0.6–2.1), *P* = 0.6
*Panic attacks*					
Yes No	48 659	27(7.9) 317(92.1)	21(5.8) 342(94.2)	1.4 (0.7–2.5)	0.8 (0.4–1.6), *P* = 0.5
*Manic*					
Yes No	26 681	18(5.2) 326(94.8)	8(2.2) 355(97.8)	2.56 (1.05–5.7)	1.3 (0.5–3.6), *P* = 0.5
*Mood disorders*					
Yes No	38 669	19(5.5) 325(94.5)	19(5.2) 344(94.8)	1.1 (0.5–2)	0.5 (0.2–1.2), *P* = 0.13
*Suicidality*					
Yes No	93 614	54(15.7) 290(84.3)	39(10.7) 324(89.3)	1.5 (0.99–2.4)	1.2 (0.7–2), *P* = 0.6
*Depression*					
Yes No	101 606	63 (18.3) 281(81.7)	38 (10.53) 325(89.5)	1.9 (1.2–3)	1.7 (0.96–3), *P* = 0.06

## Discussion

The present study emphasizes the high prevalence of mental illness among detainees in French Guiana [9]. Overall, 512/707 inmates (72%) presented at least 1 psychiatric disorder. The most common diagnoses were antisocial personality disorder (34.6%), substance addiction (33.2%), generalized anxiety disorder (25.7%), alcohol addiction (17.5%), post traumatic stress disorder (15.1%), and major depression (14.3%) (Fig. 3).

Prevalence of psychosis was greater in our sample (7.6%) than in large meta-analyses (3.7–4%) [8, 13]. Prevalence of depression was also higher. This may reflect the use of the MINI, rather than the use of other diagnostic instruments in other studies in the literature. Although antisocial personality disorder was the most frequent diagnosis, its prevalence was less frequent than in the largest meta-analysis of cross-sectional prevalence rates of mental illness among prisoners internationally [13]. It has been observed that in low to middle income countries the prevalence of psychosis and major depression is higher than in high income countries. French Guiana, despite being a French territory thus has a profile that is closer to low and middle income countries [8]. In low and middle income countries, lower budgets for psychiatric care of psychoses may lead to a shift from mental health care towards incarceration. Another hypothesis pertains to different social and cultural norms regarding mental illness, and possible poor legal representations of mentally ill patients in the justice system [8].

When comparing our results with the study in French prisons, which used the MINI and examination by a psychiatrist, there were some differences between mainland France and the French territory of French Guiana. A striking difference was the weight of addictions in French Guiana (33.2% for drugs and 17.5% for alcohol) relative to mainland France (8.9 and 8.7%, respectively). This level of addictions was comparable to what is observed in neighboring Brazil [14, 15]. PTSD and generalized anxiety seemed more frequent in French Guiana than in mainland France (15.1 and 25.7%, versus 6.6 and 15.4%) whereas depression was less frequent in French Guiana than in mainland France (14.3 versus 22.9%). Some of these differences may be due to methodological differences, but others may be due to the “Latin American socio economic and cultural context” in French Guiana. Thus what applies in mainland France may not apply in French Guiana, and French-trained mental health professionals require some adaptation of their epidemiological assumptions.

After adjusting for age, males, antisocial personality disorder, substance and alcohol addictions, depression and psychoses were significantly associated with repeat offenses as described elsewhere [16, 17]. We did not find any association with bipolar disorder. It may be that our sample size was insufficient to detect any effect because the crude odds ratio, but not the adjusted one, was significantly associated with repeat incarceration. The calculation of population-attributable fractions is arguable in this cross-sectional design. It was meant to estimate, what proportion of repeat offenses could be attributed to the psychiatric conditions, and thus what could theoretically be gained if they were controlled. We believe it emphasizes the importance of enhancing psychiatric and social care in prison.

The variables significantly associated with incarceration for violent crimes were male sex, psychosis, suicide risk. Generalized anxiety disorder on the contrary was associated with a lower probability of incarceration for a violent crime. The direction of causality is not clear with regard to suicide risk: perhaps remorse explained the association, but hetero-aggressive behavior in persons with suicidal thought is also a possible explanation. We have previously described predictive factors for suicide risk for this population [9].

Substance addiction has been reported to be associated with violence [18]. However, here we did not find any significant association perhaps because violence between drug users may be less frequently reported to the police and less frequently prosecuted. Prison is likely to represent an opportunity to initiate addiction treatment and medical services that should aim for a smooth transition with the services outside of prison [19, 20].

The transinstitutionalization hypothesis posits that mentally ill persons without proper care will be over-represented in the criminal justice system. This hypothesis is debated. Some authors argue that this is reductionist and that psychiatric desinstitutionalization does not necessarily lead to a direct increase in the number of prisoners with mental health issues, and that increasing psychiatric facilities will not necessarily reduce numbers of detainees with mental health issues [7, 21–25].

French Guiana has a structural lag in health care personnel, notably in terms of specialized physicians. In terms of the density of psychiatrists French Guiana is six times lower than in mainland France [26]. The number of beds per capita is much lower than in mainland France and even than other French overseas territories [27]. In terms of psychiatric care, the number of beds per 100,000 persons in French Guiana is lower than in other territories (90 in France, in Martinique, 70 in Guadeloupe, and 40 in French Guiana). When we compare the incarceration rate and the psychiatric facilities, the contrast between French territories is even more salient: in France there are 1.12 inmates per psychiatric bed, in Guadeloupe 3, in Martinique 3.3 and in French Guiana 8.2.

It is noteworthy that in early 2017, following a wave of violent crime, French Guiana erupted in mass protests over notably security issues and deficient health infrastructures [28]. When looking at the very high prevalence of psychiatric disorders observed in our sample, and when looking at the relative lack of psychiatric facilities, it is tempting to hypothesize that part of the problem can be explained by transinstitutionalization and the Penrose hypothesis [21, 29]. At least, we hope these data will help fuel strategic debates in French Guiana.

There are several limitations to our study: The cross sectional design and the use of declarative data in a context of incarceration may have led to biased estimates. The MINI scale is a validated screening tool but it may not always be sufficiently sensitive notably for certain borderline personality disorders which are associated with incarceration. Finally the inclusion of study participants directly after incarceration– a stressful period- may have impacted responses to the questions.

## Conclusions

Mentally ill detainees should benefit from effective screening, case identification and psychiatric care on arrival [9], ongoing care during the period of incarceration and pre-release arrangements for ongoing care in the community following release from custody. The goals of the correctional facilities and the psychiatric system are different but they may not always be antagonistic. Improved recognition and care of persons with mental illness in prison settings may improve outcomes for affected individuals and for public security [30].

## Abbreviations

CEEI: Comité d’Evaluation Ethique de L’INSERM
CNIL: Commission Nationale Informatique et Libertés
GDP: Gross Domestic Product
HIV: Human immunodeficiency virus
INSERM: Institut National de la Santé et de la Recherche Médicale
UCSA: Unité Carcérale de Soins Ambulatoires
